# Maximum likelihood estimation of spatially dependent interactions in large populations of cortical neurons

**DOI:** 10.3389/fncom.2025.1639829

**Published:** 2025-08-13

**Authors:** Camille Godin, J. P. Thivierge

**Affiliations:** ^1^School of Psychology, University of Ottawa, Ottawa, ON, Canada; ^2^Brain and Mind Research Institute, University of Ottawa, Ottawa, ON, Canada

**Keywords:** distance dependent connectivity, maximum likelihood, visual cortex (V1), calcium imaging, spiking neuron model

## Abstract

Understanding how functional connectivity between cortical neurons varies with spatial distance is crucial for characterizing large-scale neural dynamics. However, inferring these spatial patterns is challenging when spike trains are collected from large populations of neurons. Here, we present a maximum likelihood estimation (MLE) framework to quantify distance-dependent functional interactions directly from observed spiking activity. We validate this method using both synthetic spike trains generated from a linear Poisson model and biologically realistic simulations performed with Izhikevich neurons. We then apply the approach to large-scale electrophysiological recordings from V1 cortical neurons. Our results show that the proposed MLE approach robustly captures spatial decay in functional connectivity, providing insights into the spatial structure of population-level neural interactions.

## Introduction

Understanding the organization of brain networks is a fundamental challenge in neuroscience (Honey et al., 2010). A key principle of neuronal wiring is spatial embedding, where the probability of synaptic contact between neurons decreases as a function of physical distance (Ercsey-Ravasz et al., 2013; Horvát et al., 2016; Perin et al., 2011; Song et al., 2005; Stetter et al., 2012; Zingg et al., 2014). This principle holds across multiple spatial scales, from local neuronal networks to large-scale brain regions (Markov et al., 2013), and has been leveraged in computational models to explain various properties of cortical connectivity, neuronal communication (Ercsey-Ravasz et al., 2013), and functions including learning and working memory (Keller et al., 2024).

Advances in experimental neuroscience have enabled large-scale recordings of neural activity, capturing the dynamics of broad neuronal populations (Stosiek et al., 2003; Stringer et al., 2019a,b). These advances provide unprecedented insight into the complex interactions that govern brain function (Chen et al., 2022; Honey et al., 2010; Seth, 2008; Suárez et al., 2020). However, characterizing distance-dependent interactions within these populations remains challenging, as the underlying synaptic connectivity is typically unknown *in vivo* (Tian et al., 2024). Addressing this challenge would provide a rich understanding of neuronal organization and enable comparisons across brain regions that play distinct roles in behavioral and mental states.

To examine distance-dependent interactions, most studies have relied on functional connectivity to measure statistical dependencies or mutual information between patterns of neuronal activity (Friston et al., 2013; Stetter et al., 2012). While measuring structural connectivity requires access to the physical connections between neurons, based on invasive or post-mortem techniques, functional connectivity can be derived from recorded neural activity. This makes functional connectivity a pivotal approach for investigating the dynamic relationship between interconnected neural elements. Thus, estimating functional connectivity is critical for understanding information flow in the brain and the organization of neuronal circuits.

A variety of measures have been proposed to infer functional connectivity, including pairwise correlations, transfer entropy, and Granger causality (Tian et al., 2024). Distance-based interactions estimated from pairwise correlations (Smith and Kohn, 2008; Yatsenko et al., 2015) and spike-based functional connectivity (English et al., 2017) have been measured in cortex. These interactions show specificity across layers (Senzai et al., 2019), cell types (Khoury et al., 2025), brain regions (Horvát et al., 2016), as well as a dependence on sensory features such as stimulus orientation (Smith and Kohn, 2008; Yatsenko et al., 2015).

However, measures of functional connectivity have notable limitations. First, estimating functional connectivity is complicated by the inherently noisy and irregular nature of neuronal signals (Aertsen et al., 1989; Ito et al., 2011; Izzi et al., 2024; Liao et al., 2010; Vicente et al., 2011). Second, functional connectivity does not directly measure synaptic efficacy, making interpretation difficult (Ursino et al., 2020). Third, conventional functional connectivity measures do not explicitly account for spatial interactions, often overlooking the impact of physical distance on neuronal communication (Holmgren et al., 2003; Jiang et al., 2015; Perin et al., 2011; Vegué et al., 2017). As a result, these methods fail to capture the underlying spatial structure of neuronal networks, limiting our ability to understand their functional organization. Given the critical role of spatially structured interactions in neural processing (Ohiorhenuan et al., 2010), new approaches are needed to extract meaningful spatial information from large-scale recordings.

Here, we introduce a novel maximum likelihood estimation (MLE) approach to characterize distance-dependent functional connectivity. Instead of estimating individual interaction strengths between all pairs of neurons, this method estimates a global parameter that governs the spatial decay of connectivity across a neuronal population. The proposed MLE assumes an exponential relationship between connectivity and physical distance (Ercsey-Ravasz et al., 2013; Horvát et al., 2016) and infers this decay parameter from observed spike trains.

We validate this approach using synthetic networks of Poisson and Izhikevich neurons (Izhikevich, 2003) and apply it to large-scale recordings from mouse V1 neurons obtained from two-photon calcium imaging (Stringer et al., 2019a). Our results demonstrate that MLE successfully extracts distance-dependent interactions, revealing spatially structured neuronal communication.

## Results

### Distance-based interactions in a linear Poisson model

**FIGURE 1:**
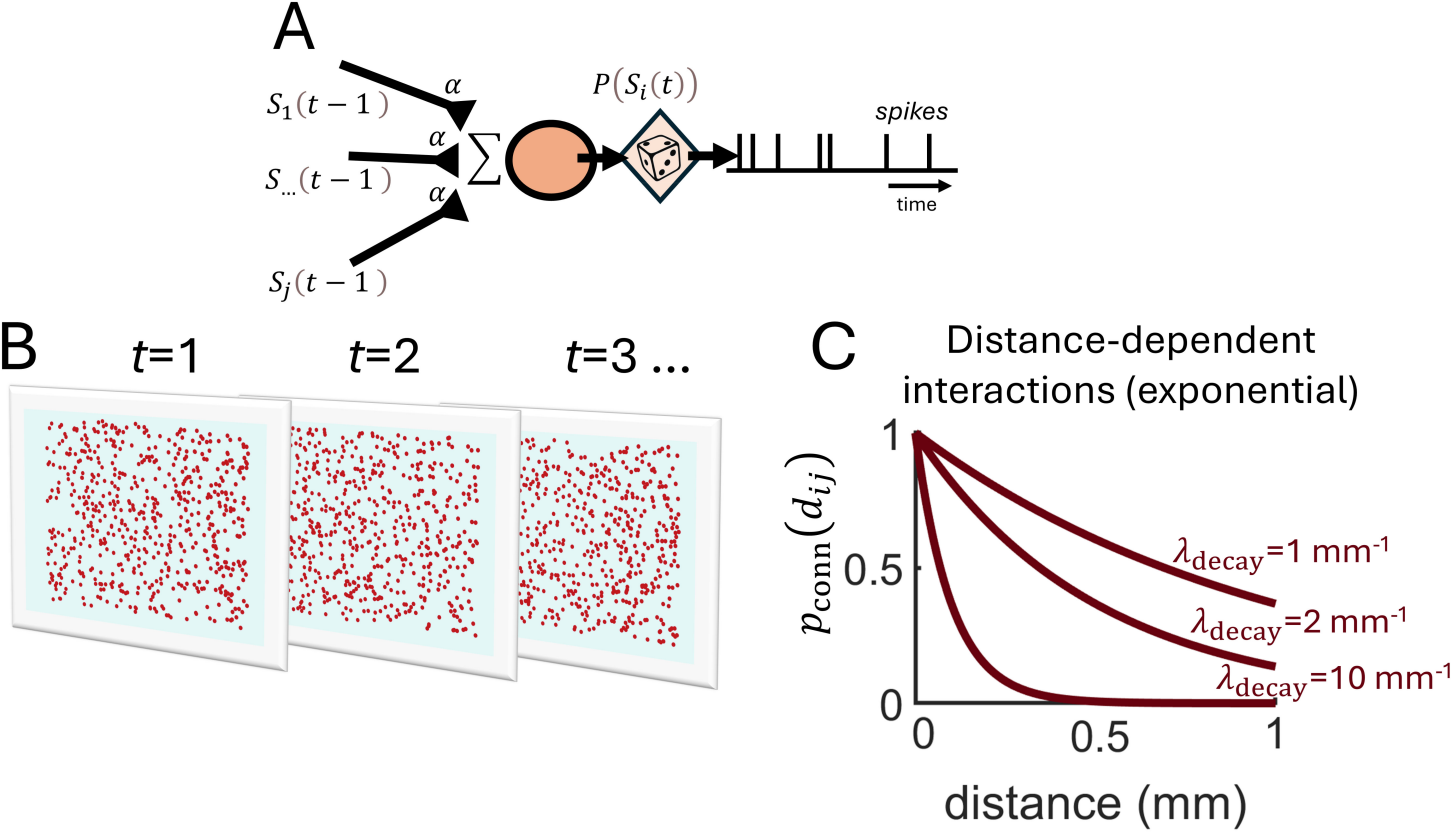
Spatially embedded Poisson neurons. **(A)** Schema of a stationary Poisson neuron receiving spiking activity *S*_*j*_(*t*−1) from *j^th^* presynaptic neurons at time *t-1* via weighted connections α. Presynaptic activity is summed and a stochastic process generates spikes *p*(*S*_*i*_(*t*)). **(B)** Illustration of spiking activity over time-steps *t* for a population of neurons randomly positioned on a two-dimensional grid. **(C)** The probability of connection between pairs of neurons is determined by an exponential function with slope parameter λ_decay_.

As a starting point, we designed a simple linear model where spike trains from individual neurons are generated by stationary Poisson processes, implying that the number of spikes in non-overlapping intervals is conditionally independent and that the spike rate remains constant over time (Figure 1A) (see section “Materials and methods”). The spike probability *p*(*S*_*i*_(*t*)) of a neuron *i* at time *t* depends on the baseline rate (*r_0_*) and the spiking activity of surrounding neurons,


$$p\,\left(S_{i}\,\left(t\right)\right)=r_{0}+\sum_{j}\left(J_{i\,j}\cdot \alpha \cdot S_{j}\,\left(t-1\right)\right)$$

where *S*_*j*_(*t*−1) is the spiking activity of neuron *j* at the previous time-step, α is a fixed coupling strength, and *J*_*ij*_ = 1 if neuron *j* is connected to neuron *i*, and 0 otherwise, where the connection probability follows an exponential function with a decay parameter (λ_decay_) (Figure 1C),


(1) $$\mathbb{P}\left(J_{i\,j}=1\right)=exp\left(-\lambda_{\text{decay}}d_{i\,j}\right)$$

where *d*_*ij*_ denotes the Euclidean distance between neurons *i* and *j*, expressed in units of millimeters. In this model, neurons are recurrently connected and embedded in two-dimensional space, meaning that each neuron is assigned a unique position along a finite two-dimensional grid where boundaries do not wrap around. Hence, spiking activity in this model is described by both spatial and temporal coordinates (Figure 1B).

**FIGURE 2:**
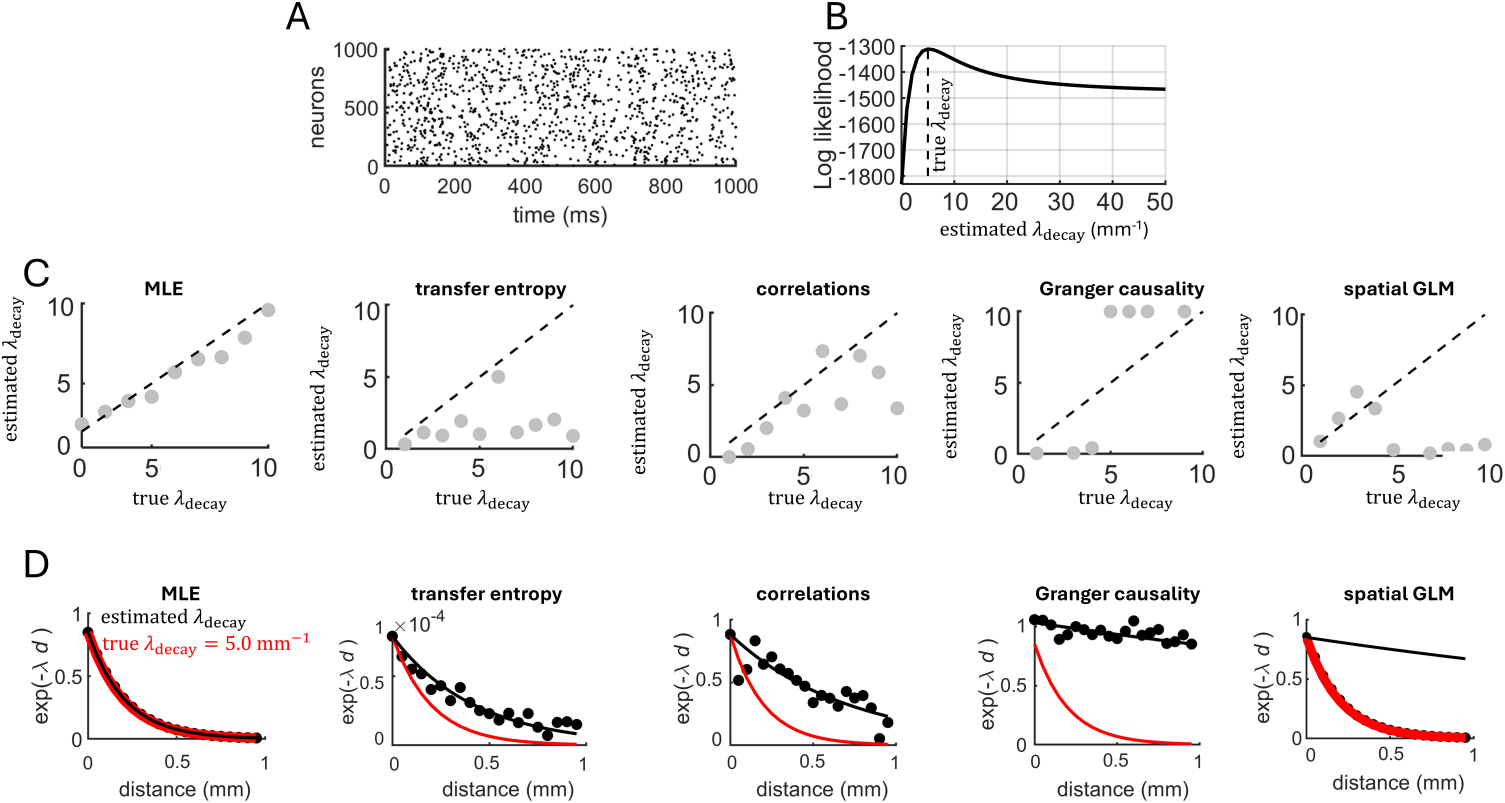
Estimating spatially dependent interactions in a network of Poisson neurons. **(A)** Spike raster generated by 1,000 interconnected neurons. **(B)** The log likelihood function was maximal near the true value of λ_decay_. **(C)** The maximum likelihood estimate of λ_decay_ across different runs of the Poisson model. Dashed line: unity line showing the correspondence between the true and estimated values of λ_decay_. Five approaches were employed to estimate λ_decay_, from left to right: MLE, transfer entropy, pairwise correlations, Granger causality, and spatial GLM. **(D)** Estimated value of exponential decay. Filled black circles are individual data points obtained from connection probability (MLE and spatial GLM), transfer entropy, spike-count correlations, Granger causality.

An example of a spike raster generated with this model using λ_decay_ = 5 mm^–1^ is shown in Figure 2A (1 time-step set as equivalent to 1 ms). The central question addressed here is whether we can reliably estimate λ_decay_ based on this spike raster. This was performed by maximum likelihood estimation (see section “Materials and methods”). Because λ_decay_ is estimated from spike trains and not synaptic connections, it captures the functional interactions between spatially embedded neurons, and is not a proxy for physical connectivity, though the two may be linked, as exemplified below.

A numerical example of the log likelihood obtained from 1,000 Poisson neurons simulated for 100 s of activity is shown in Figure 2B. The log likelihood was maximal near the true value of λ_decay_ = 5 mm^–1^. A similar result held across a range of λ_decay_ values (Figure 2C). Hence, MLE yielded a close approximation of the λ_decay_ parameter, providing a characterization of distance-dependent interactions based on the neural data. MLE also approximated distance-dependent interactions characterized by half-Gaussian, linear, and lognormal functions, albeit poorer results were obtained with an inverse square (Supplementary Figure 1).

To evaluate the extent to which spike data from the Poisson model could be used to recover underlying distance-dependent functions, we applied a spatial Generalized Linear Model (GLM, see section “Materials and methods”) (Pillow et al., 2008). Five different generating functions were employed to produce spike trains (exponential, half-Gaussian, linear, inverse square, and lognormal). The maximum likelihood of each generating function was computed to identify the most likely candidate function. Results show that the exponential, half-Gaussian, linear, and inverse square functions could be identified based on their maximum log likelihood (Table 1). The lognormal function, however, was confounded with the linear function and was therefore excluded from further analyses. Thus, maximum likelihood estimation based on a spatial GLM enabled the identification of several distance-dependent functions that determined spatial interactions in the Poisson model.

**TABLE 1:** Identification of various distance-dependent functions.

Generating function	Candidate function				
	Exp.	Half-Gaussian	Linear	Inv. square	Lognormal
Exponential	**−5998.49**	**−**7930.84	**−**11767.10	**−**615020.87	**−**379343.05
Half-Gaussian	**−**795.60	**−794.79**	**−**796.48	**−**797.38	796.91
Linear	**−**987.77	**−**10687.99	**−7417.71**	**−**94061.43	**−**79478.56
Inverse square	**−**918.71	**−**918.89	**−**919.00	**−915.27**	**−**916.43
Lognormal	**−**851.28	**−**851.44	**−850.95**	**−**852.80	**−**852.64

For each row, the highest log-likelihood across candidate functions is shown in bold.

### Comparison to related approaches

Simulation results obtained with MLE were compared to three related approaches. First, functional connectivity between all pairs of simulated neurons was computed using transfer entropy (Kobayashi and Shinomoto, 2024; Vicente et al., 2011). A least-squares approximation was employed to find the best-fitting value of λ_decay_ relating transfer entropy to spatial distances between pairs of neurons. This approach offered a poor fit to the true value of λ_decay_ from which spike trains were generated (Figure 2C).

In a second approach, spike-count correlations were computed between all pairs of neurons using non-overlapping time bins (10 ms), then least-squares was applied to find the λ_decay_ that best describes the relation between correlations and spatial distances. This approach offered a marginally better fit than transfer entropy, but its accuracy remained lower than MLE. Third, Granger causality (Tian et al., 2024) was computed followed by least-squares fitting, yielding a poor fit between neural interactions and spatial distances. A fourth approach employed the spatial GLM to estimate values of λ_decay_, again yielding a poor fit compared to the MLE. Examples of fit obtained from MLE, transfer entropy, Granger causality, spatial GLM and spike-count correlations are shown in Figure 2D. In summary, the fit obtained with MLE was not attained with the alternative approaches.

**FIGURE 3:**
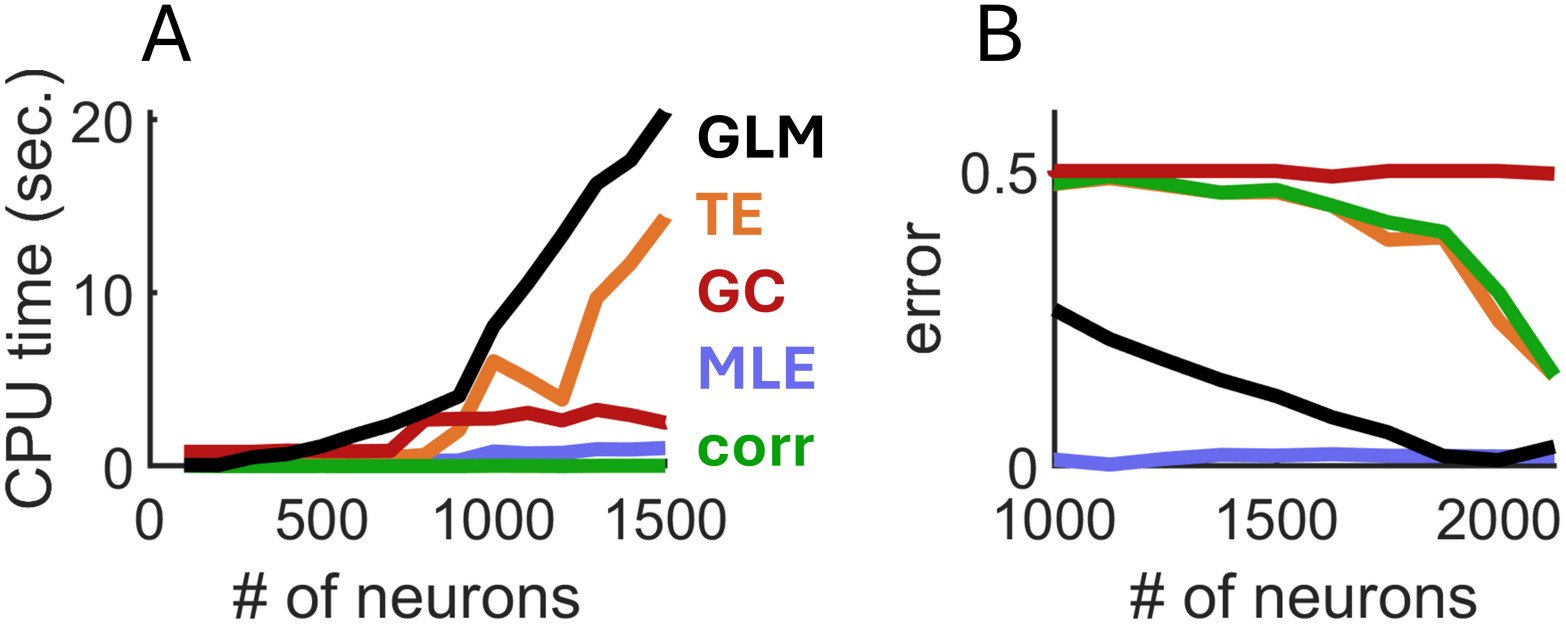
Comparison of run time **(A)** and mean error **(B)** of four approaches to estimate exponential spatial decay. GLM, generalized linear model; TE, transfer entropy; GC, Granger causality; corr, spike-count correlations.

Next, we examined the computational run time required for MLE, transfer entropy, Granger causality, spatial GLM, and spike-count correlations. Run times were computed on an Intel i9 processor at 2500 Mhz. For small networks consisting of 100–500 neurons, run times were similar across the five approaches (Figure 3A). However, as the number of neurons increased, the run time of transfer entropy and spatial GLM increased rapidly, whereas the runtimes of MLE, Granger causality, and spike-count correlations remained comparatively low. Overall, MLE required only marginally higher run time than spike-count correlations.

In follow-up analyses, we compared the various approaches in terms of estimation error (computed as the absolute difference between the true and estimated values of λ_decay_). We fixed λ_decay_ = 5 mm^–1^ and systematically increased the number of simulated neurons. MLE attained markedly lower error compared to related approaches **(**Figure 3B). While the spatial GLM achieved similar performance to MLE with >1,500 neurons, this came at the cost of a high CPU time. Thus, MLE provided an accurate estimation of spatial interactions based on spiking activity and required conservative run times compared to alternative approaches.

Additional analyses revealed that while several factors impacted MLE accuracy, including firing rates, subsampling, and synaptic delays, error remained low under typical experimental conditions (Supplementary Figures 2–4).

### Izhikevich model

**FIGURE 4:**
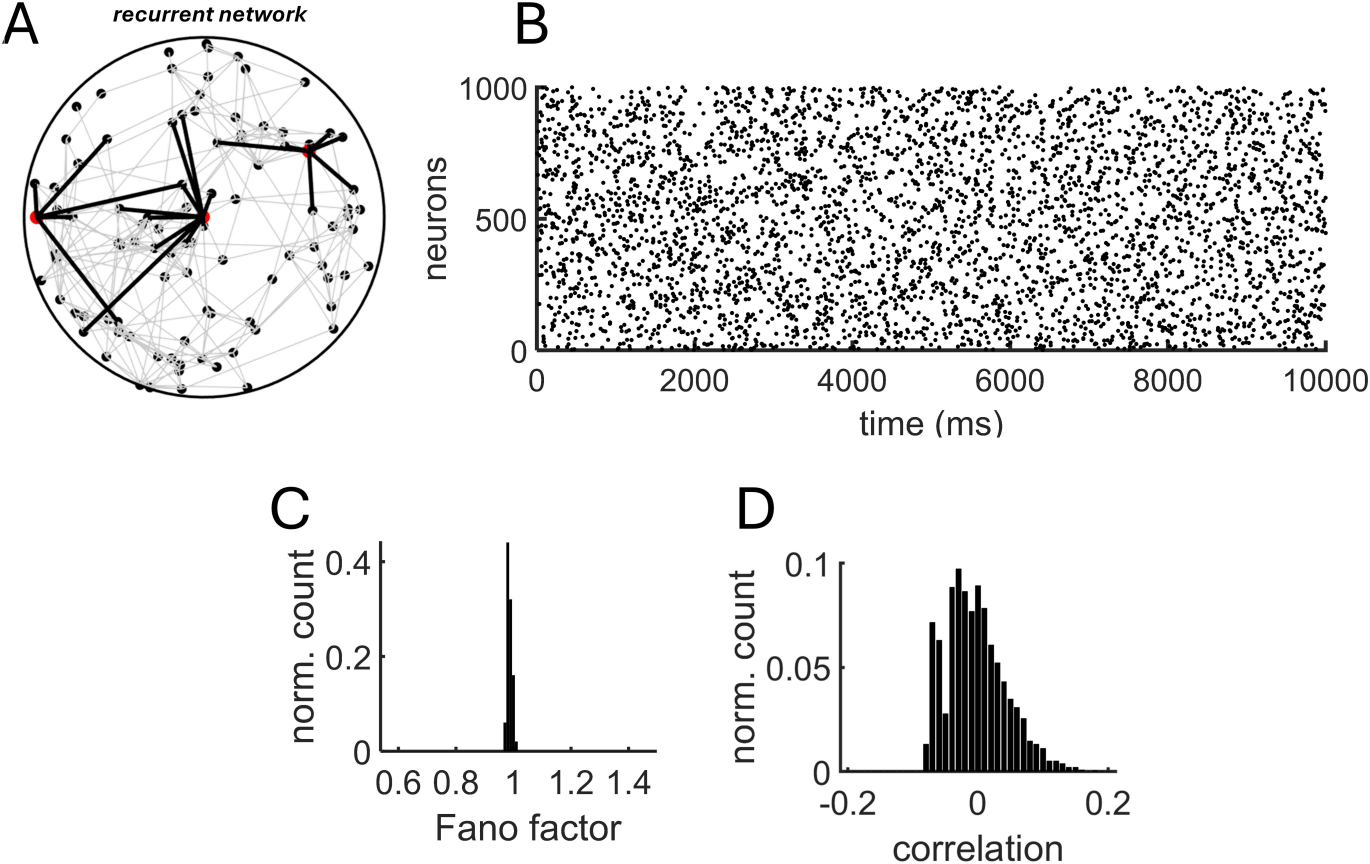
Recurrent Izhikevich model with distance-dependent connectivity. **(A)** Schema of the recurrent model. The connections (black lines) of three neurons (red circles) are highlighted to show spatially-delimited interactions with neighboring units. **(B)** Spike raster obtained from a simulation of 1,000 neurons. Distance-dependent connectivity followed an exponential distribution with λ_decay_ = 5 mm^– 1^. **(C)** Distribution of Fano factors obtained from all individual neurons using 10 ms non-overlapping bins. **(D)** Distribution of spike-count correlations.

Going beyond a linear Poisson model, we examined a network of Izhikevich neurons with distance-dependent connectivity (Figure 4A). The model generated sparse, uncorrelated activity (Figure 4B). Importantly, because the MLE approach assumes that spike trains are close to Poisson statistics, we computed the Fano factor (FF) – the ratio of spike count variance to its mean – for all neurons and ensured a distribution with mean FF ∼ 1 (Figure 4C). In addition, the mean spike-count correlation across all pairs of neurons was close to zero (mean: 0.0017) (Figure 4D).

**FIGURE 5:**
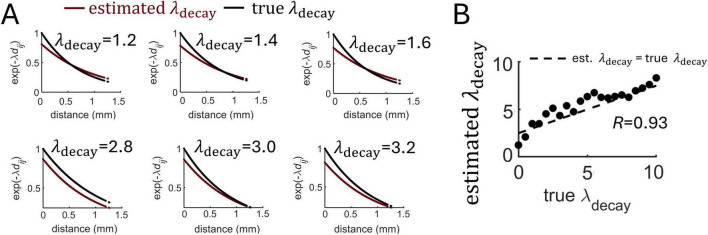
Estimation of distance-dependent connectivity using MLE. **(A)** Examples of exponential functions estimated from different runs of the Izhikevich model that varied the true value of λ_decay_. **(B)** Estimation of λ_decay_ across various runs of the model. Dashed line: best-fitting regression.

To test the proposed MLE approach, a series of simulations were performed where a population of 1,000 neurons generated spike trains for 100 s with varying values of λ_decay_. Examples of fit between the true and estimated λ_decay_ are shown in Figure 5A. Close correspondence between these values was found over a range of parameters (Figure 5B).

**FIGURE 6:**
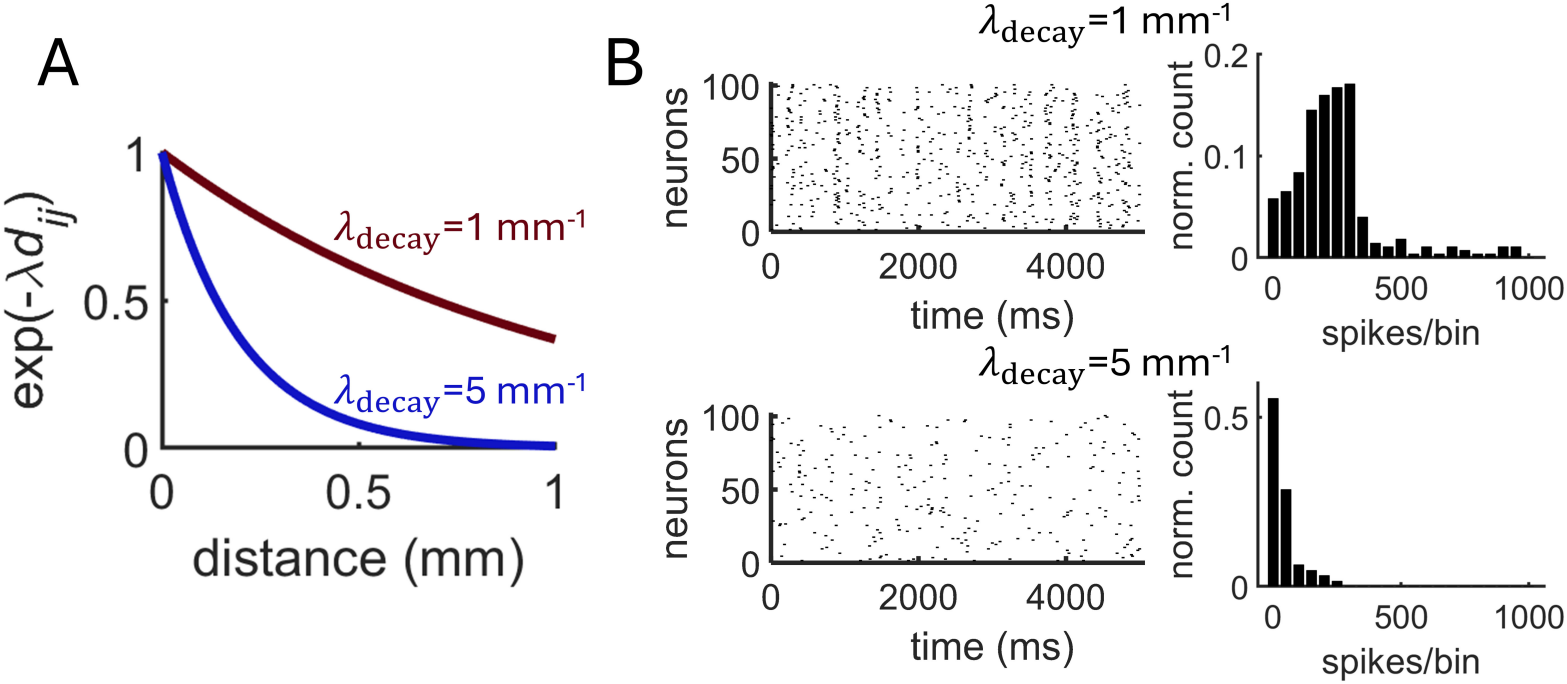
Impact of distance-dependent connectivity on population dynamics in the Izhikevich model. **(A)** Two exponential decay functions tested on the model. **(B)** Spike rasters (left) and distribution of synchronous spikes using 10 ms bins (right) obtained with two different decay functions.

**FIGURE 7:**
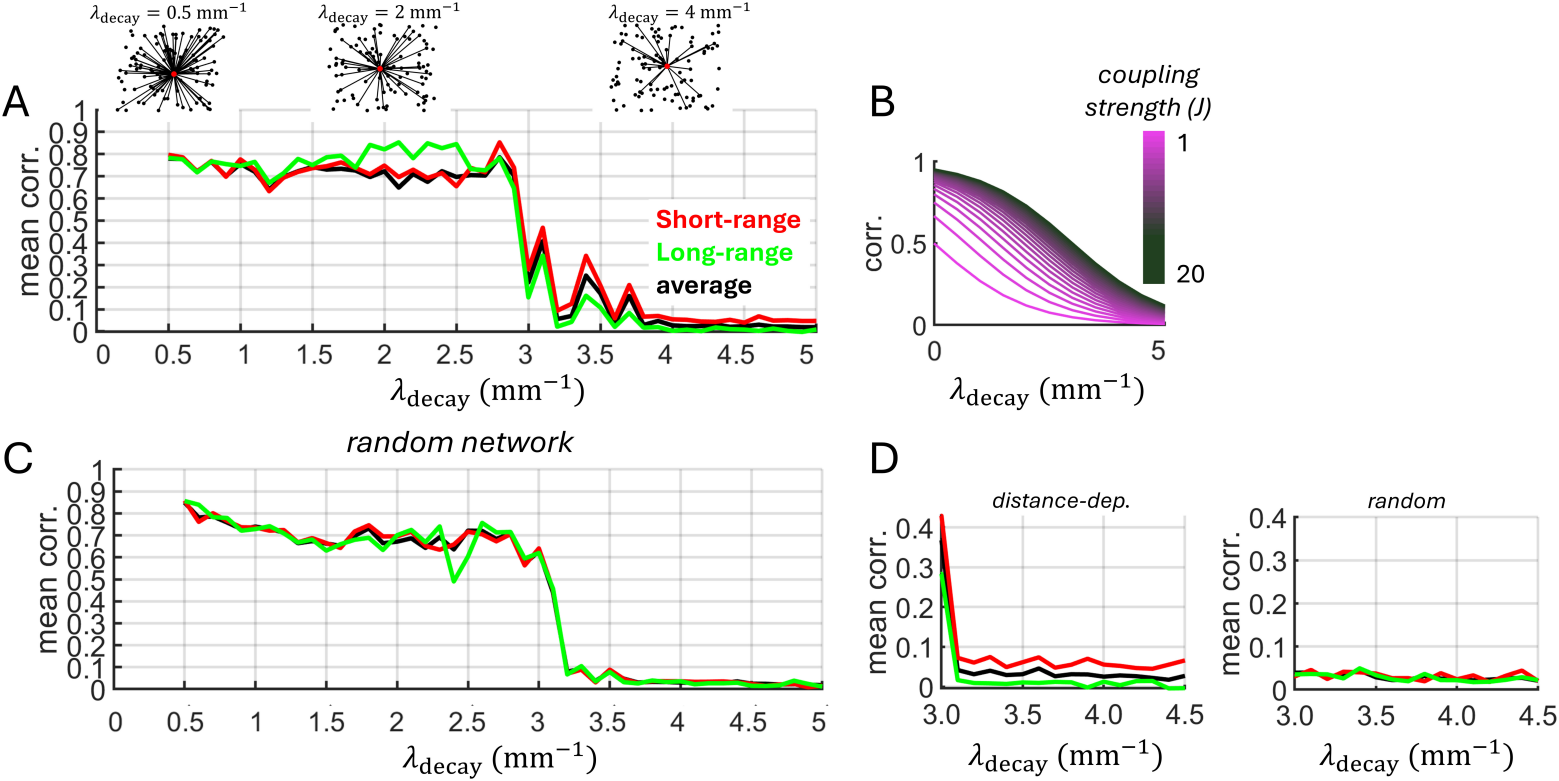
Spike-count correlations are modulated by distance-dependent connectivity. **(A)** Exponentially decaying connectivity modulates correlations. Above: schema of connectivity patterns. Short-range: mean of correlations between neurons with distance <0.1 mm. Long-range: neurons with distance >1 mm. Average: mean over all neurons. **(B)** Analytical expression relating correlations to spatial decay and coupling strength (Equation 2). **(C)** Randomized network obtained after shuffling weights from panel “A.” **(D)** Zoom of panels “A” and “C” for higher values of λ_decay_.

Next, the Izhikevich model was employed to investigate the impact of distance-dependent interactions on neural dynamics. Broadening interactions by increasing λ_decay_ from 1 mm^–1^ to 5 mm^–1^ (Figure 6A) resulted in a decrease in synchronized activity (Figure 6B). This makes intuitive sense given that longer-range connectivity promotes communication between a broader network of neurons. In turn, broadening spatial interactions by lowering λ_decay_ in a range between 0.5 and 3.0 resulted in a high average spike-count correlations (Figure 7A).

Interestingly, an abrupt transition was observed between a state of high and low correlations, occurring around λ_decay_ = 3.0. To formally examine this transition, we considered an approximation for the correlation *C*_*ij*_ between pairs of neurons receiving a shared input under the assumption of weak coupling (see section “**Materials and Methods”**),


(2) $$C_{i\,j}\approx \frac{J^{2}}{J^{2}+\sigma^{2}}\,e^{-2\,\lambda_{\text{decay}}\,d_{i\,j}}$$

where *J* is the mean synaptic strenght and σ^2^ is the noise variance of the shared input. Plotting Equation 2 across values of λ_decay_ shows a non-linear relation between spatially dependent connectivity and pairwise correlations (Figure 7B). Thus, particularly for stronger coupling strength *J*, correlations remained high for low values of spatial decay, with a sharp decline as decay increased further.

We further examined this effect in a scenario with randomized connectivity by shuffling the distance-dependent connections of the Izhikevich model. A similar transition in correlation was observed in the random model (Figure 7C). However, in the distance-dependent model, shorter-range correlations (< 0.1 mm) retained higher values than longer-range connections (> 1 mm) following the transition. This effect did not arise in the random model due to the lack of spatial dependencies (Figure 7D). Thus, while an abrupt shift in correlation may be explained by synaptic density rather than spatial dependency (Roxin et al., 2004), the phase transition exhibited a distance-dependent effect that was not present in the randomized model.

### Application of MLE to calcium imaging data

We applied MLE to estimate spatial interactions in two-photon calcium imaging data (Stringer et al., 2019a). These data are particularly challenging given that they are composed of approximately 10,000 neurons, making approaches based on pairwise computations prohibitive. Instead, the approach taken by MLE is to estimate the best-fitting λ_decay_ on the basis of the spike raster and the physical distance amongst neurons, without needing to compute the strength of interactions between all pairs of neurons. Thus, this approach differs from attempts at reconstructing connectivity based on calcium data (Orlandi et al., 2014; Stetter et al., 2012). In our case, the goal is strictly focused on estimating the decay parameter of a distance-based function, without reconstructing the full connectivity.

**FIGURE 8:**
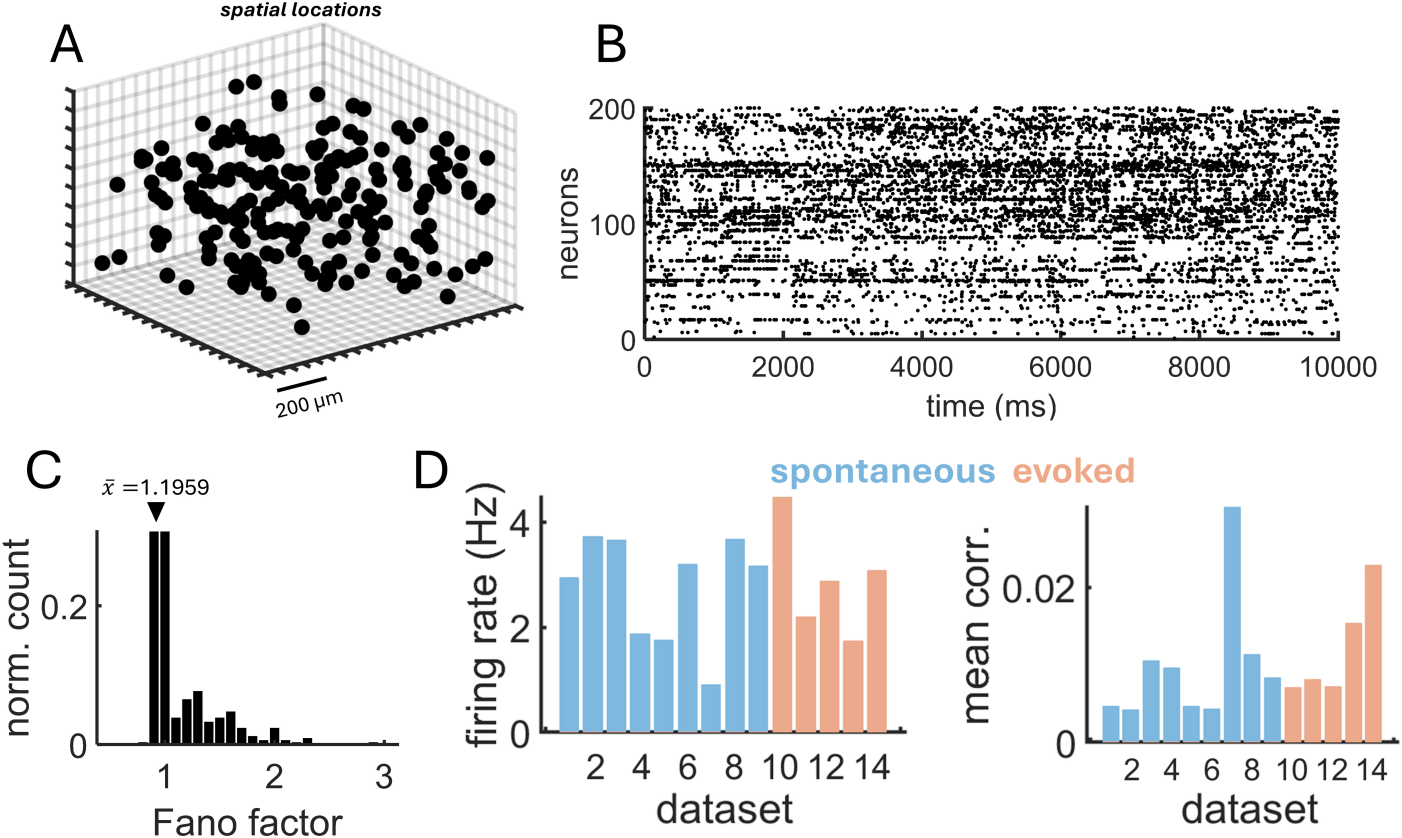
Two-photon calcium imaging data from rodent visual cortex. **(A)** Three-dimensional position of individual neurons from a subset of the population. **(B)** Raster obtained from calcium imaging data (subset of neurons from panel “A”). **(C)** Distribution of Fano factors, with mean indicated by the arrow. **(D)** Mean firing rate (left) and spike-count correlations (right) across 9 datasets of spontaneous activity and 5 datasets of evoked activity.

A total of 14 datasets were analyzed, including 9 rasters of spontaneous activity and 5 rasters of evoked activity with drifting gratings (see section “Materials and methods” for details). The mean number of neurons was 11,602. A sample of neurons arrayed according to their three-dimensional coordinates is shown in Figure 8A, showing a spatially homogeneous distribution of cell locations. A raster of spiking activity for this sample shows a weak degree of coordination across neurons (Figure 8B) with a Fano factor near FF ∼ 1, indicative of approximate Poisson statistics (Figure 8C). Mean firing rates were similar for spontaneous (2.76 Hz) and evoked (2.87 Hz) activity (Figure 8D). Mean spike-count correlations (100 ms non-overlapping time bins) were low for both spontaneous (*C* = 0.0072) and evoked (*C* = 0.0121) activity.

**FIGURE 9:**
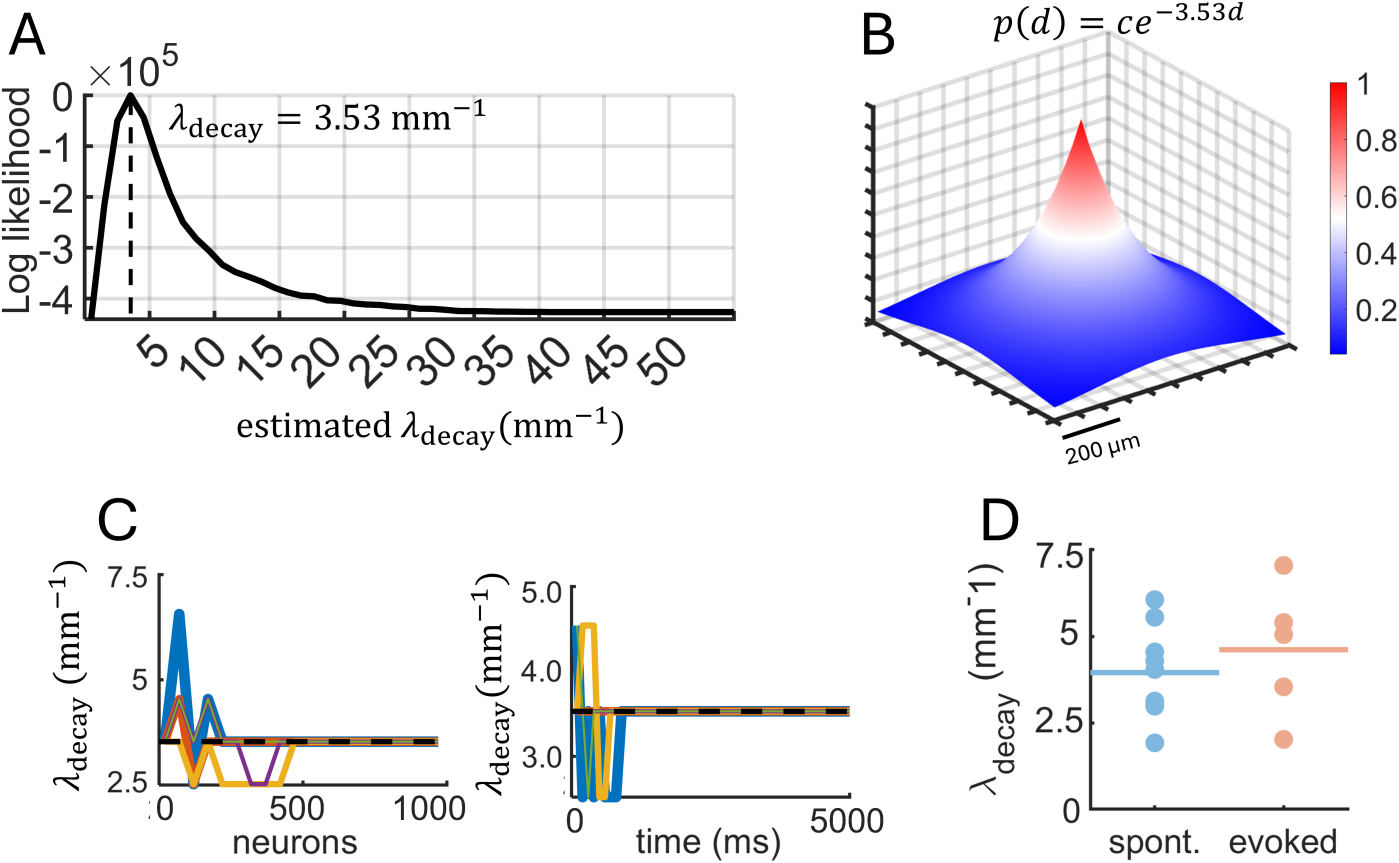
Maximum likelihood estimation (MLE) applied to two-photon calcium data. **(A)** Log-likelihood function obtained from a single raster of spontaneous activity (10,164 neurons, 15,761 ms). Vertical dashed line shows the optimal value of λ_decay_ obtained with MLE. **(B)** Best-fitting exponential function based on the log-likelihood from panel “A” shown on a two-dimensional spatial grid. **(C)** Fluctuations in the estimated λ_decay_ as the number of neurons and time increased. Dashed line: λ_decay_ value obtained with all neurons and time-steps included. **(D)** Estimates of λ_decay_ across different recording sessions of spontaneous and stimulus-evoked activity. Filled circles are individual sessions; horizontal lines are within-group averages.

An example of a log-likelihood function obtained from MLE applied to a single raster is shown in Figure 9A. The function shows a defined peak at λ_decay_ = 3.53 mm^–1^. Hence, the characteristic length scale of spatial interactions is 3.53^–1^ = 0.28 mm, which means that the probability of pairwise interactions decays significantly over approximately 0.28 mm (280 μm). To illustrate this result, we considered a two-dimensional neuronal space and assumed a putative neuron located in the center of this space. We then plotted an exponentially-distributed probability of pairwise interactions according to the function *p*(*d*) = *ce*^−3.53*d*^ (Figure 9B). This function shows that most of the interactions are contained within a delimited spatial area surrounding each neuron, in line with work showing that a majority of cortical interactions occur within a 100–300 μm radius (Ercsey-Ravasz et al., 2013; Holmgren et al., 2003; Horvát et al., 2016; Levy and Reyes, 2012; Markov et al., 2013). This result is consistent with distance-dependent interactions observed in related measures including spike-count correlations, transfer entropy, and Granger causality for spontaneous and evoked activity (Supplementary Figure 5).

Next, we examined whether the number of neurons and duration of recordings impacted the estimation of λ_decay_. Despite the large number of neurons available, a subset of ∼500 neurons was sufficient to obtain a reliable estimate of λ_decay_ (Figure 9C, left). This is encouraging in terms of applying MLE to smaller datasets. Similarly, stable estimates were obtained with less than 5,000 ms of data (Figure 9C, right). This result is likely impacted by the stationarity of spike trains, with more data points required in datasets that exhibit complex fluctuations such as metastable states, a phenomenon that is not explored here.

We found that estimates of λ_decay_ overlapped across datasets of spontaneous and evoked activity (Figure 9D). This result is consistent with an exponential decay in spatial interactions that reflects structural properties of neuronal circuits and not fluctuations across different dynamical states. Simulations of the Izhikevich model showed that restricted spatial interactions (λ_decay_ = 3 mm^–1^) result in low spike-count correlations (Figure 7A). This prediction agrees with cortical recordings, where the mean λ_decay_ was high for both spontaneous (λ_decay_ = 3.49 mm^–1^) and evoked (λ_decay_ = 4.12 mm^–1^) activity. Further, across all datasets, a negative correlation was found between values of λ_decay_ and mean spike-count correlations, showing that spatially restricted interactions yielded lower spike-count correlations (*N* = 14, *R* = −0.5477, *P* = 0.0527). Interestingly, simulated activity with values of λ_decay_ between 3–4 mm^–1^ puts the Izhikevich model at the edge of a phase transition between high and low spike-count correlations. In this state, subtle changes in distance-based interactions can have a drastic effect of neuronal dynamics.

**FIGURE 10:**
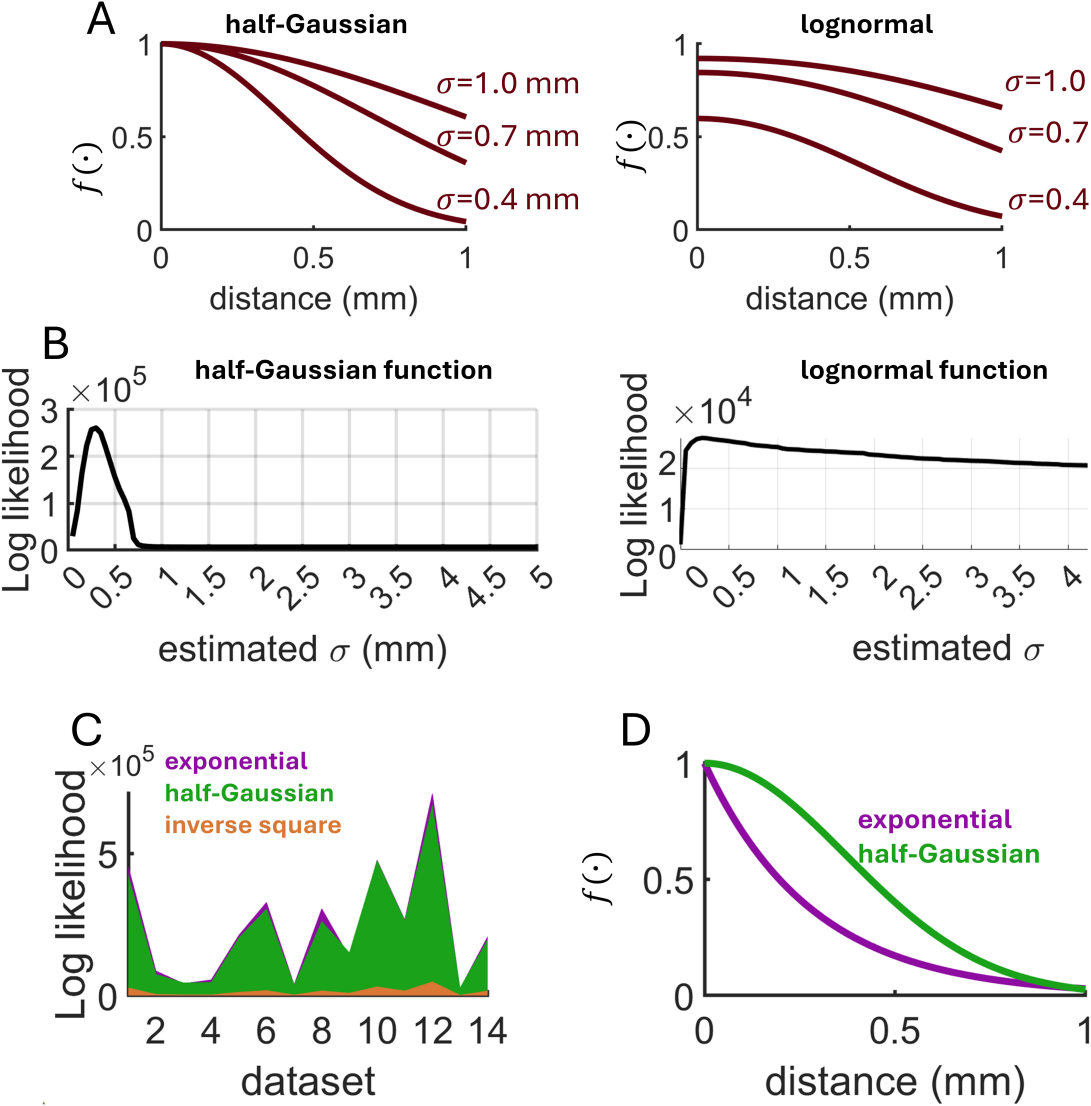
Comparison of MLE across distance-dependent functions. **(A)** Examples of half-Gaussian and lognormal functions (μ = 1.5) characterizing distance-based interactions. **(B)** Examples of log likelihood estimates obtained on single calcium imaging datasets. **(C)** Comparison of log likelihood across three candidate functions and 14 experimental datasets. Values shown were obtained by subtracting the minimum log likelihood. **(D)** Average best-fitting exponential (λ_decay_ = 3.8 mm^– 1^) and half-Gaussian (σ = 0.37 mm) functions across all experimental datasets.

Finally, we compared the fit of an exponential decay to a half-Gaussian and an inverse square decay (Figure 10A). Log likelihood estimates for these functions were derived across all 14 datasets using the spatial GLM (Materials and Methods) (Figure 10B). Overall, the exponential decay attained a higher log likelihood, indicative of a superior fit (Figure 10C). Of the 14 datasets analyzed, 5 yielded a higher log-likelihood for the half-Gaussian function (three datasets of spontaneous activity, and two evoked). When comparing the average best-fitting exponential and half-Gaussian functions, the key difference was in the steepness of the decay occurring within distances <1 mm, where the half-Gaussian function suggested a shallower decay (Figure 10D). The best fitting half-Gaussian function had a mean standard deviation across datasets of σ = 0.37, suggesting that 95% of functional interactions resided within a distance of 2σ = 0.74 mm. This value is consistent with estimates of synaptic connectivity in rodent visual cortex, where the slope of the half-Gaussian function varied between 0.49 (layer 2) and 0.3 (layer 3) (Hellwig, 2000).

Overall, these results suggest that within local circuits of the visual cortex, distance-dependent interactions are best characterized by an exponential decay. To be sure, this finding may depend on several factors not explored here. For broader spatial scales, including inter-areal interactions, the decay function would be broader to reflect longer-range connections (Campagnola et al., 2022; Ercsey-Ravasz et al., 2013; Horvát et al., 2016). Further, we cannot rule out the possibility that distance-based interactions reflect unique connectivity patterns across cortical layers and may be altered under different regimes of neuronal activity that reflect cognitive states (Huang et al., 2019).

## Discussion

This study introduced a statistical method based on maximum likelihood estimation (MLE) to investigate distance-dependent interactions from observed neuronal population activity. A key advantage of this approach is its ability to estimate spatial decay directly from spike data without requiring a full reconstruction of all pairwise interactions, leading to considerable savings in computational time when scaled to large networks, hence enabling the characterization of spatial interactions across large populations of neurons. Combined with simulations of spatially-embedded networks, this approach draws links between the structure and dynamics of neuronal circuits by relating spatial interactions and synchronous activity.

When applied to neuronal activity obtained from calcium imaging (Stringer et al., 2019a), MLE revealed spatially-delimited interactions that were consistent across both spontaneous and evoked states, suggesting an underlying network architecture that is strongly biased toward stronger interactions amongst nearby neurons. This result is consistent with a growing understanding that neural circuits are fundamentally shaped by their spatial embedding (Wang and Kennedy, 2016). This embedding emerges across a wide range of spatial scales, ranging from local microcircuits (Campagnola et al., 2022) to large inter-areal projections (Markov et al., 2013), and carries significant consequences for both the cytoarchitectural organization of the brain as well as the dynamical patterns of activity that emerge from this architecture.

One consequence of spatially constrained connectivity is the relative rarity of “giant hubs” characterized by neurons (or whole areas) that receive dense afferences from both nearby and distant projection sites (Deco et al., 2021). While disproportionally rare, these hubs may fulfill important functional roles in relaying information across broad networks, with implications for information processing (Ohiorhenuan et al., 2010). As such, a full understanding of cortical organization relies on several requisites, including not only distance-dependent interactions, but also hubs and locally clustered connectivity (Litwin-Kumar and Doiron, 2012).

The prevalence of an exponential decay in spatially embedded brain networks may be attributed to a trade-off: on one side, the need for efficient communication, and on the other, the energetic and spatial costs associated with axonal extension, myelination, and maintenance. Theoretical work shows that an exponential decay optimizes this trade-off in a scale invariant fashion, making spatially-embedded models a widely applicable framework for describing brain networks at various levels of organization (Ercsey-Ravasz et al., 2013; Horvát et al., 2016).

Spatially embedded interactions carry profound implications for the dynamical state of neuronal circuits, shaping the scope of activity that can be expressed at the population level. Various forms of spatiotemporal dynamics, including traveling waves (Huang et al., 2019) and spiral waves (Paraskevov and Zendrikov, 2017) are supported by spatially ordered connectivity and form a hallmark of information propagation across brain areas (Keller et al., 2024). Further, theoretical results presented here demonstrate a direct impact of distance-based interactions on spike-count correlations (Figure 7), showing a key role of spatially constrained connectivity in shaping population activity. This prediction of the model will need to be validated in experiments where the strength of distance-based interactions can be modulated, for instance by altering sensory input (Ohiorhenuan et al., 2010).

While our analyses revealed that an exponential decay function prevailed over alternatives including a half-Gaussian and a lognormal function, this result may depend upon several factors including the dynamical state of the network, the spatial scale of analysis, the laminar origin of the data, and the brain area considered (Horvát et al., 2016; Paraskevov and Zendrikov, 2017; Pastorelli et al., 2018). In particular, understanding how connectivity varies across cortical layers—with superficial layers showing more lateral, long-range connections and deep layers exhibiting more localized connectivity—could provide important insights into the organization of neural circuits (Holmgren et al., 2003; Levy and Reyes, 2012). As a caveat, candidate functions including an exponential and half-Gaussian decay may yield similar predictions at shorter spatial scales (< 1 mm) (Figure 10D).

While an MLE approach provided accurate estimates of spatially-dependent interactions in both a linear Poisson model (Figure 2C) and an Izhikevich network (Figure 5B), it is worth re-iterating sources of error that may compromise accuracy, including the number of neurons considered, duration of activity, firing rate, subsampling of neurons, spatial edge effects, domain size, and neuronal delays. Further, because MLE evaluates functional interactions and not structural connectivity, it reflects a combination of mono- and poly-synaptic interactions, and hence may overestimate the spatial extent of direct synaptic connections. In recent work, the connection probability of mouse neocortex as a function of lateral intersomatic distance was estimated by fitting a Gaussian decay with σ values ranging from between 0.1 mm and 0.13 mm (Campagnola et al., 2022). These values are lower than our findings (mean of σ = 0.37 mm), but comparable to visual cortical reconstruction in other work (Hellwig, 2000).

In general, estimates of functional connectivity inferred from spike data may serve as a proxy for synaptic connectivity when several conditions are met, including: (1) spike trains are stationary over the time window of estimation; (2) functional connectivity predominantly targets monosynaptic interactions; (3) the common input between pairs of neurons is low; (4) synaptic connectivity is sparse; (5) excitation and inhibition is balanced, to avoid high synchronization or inactivity; (6) neural dynamics are approximately linear (Cai et al., 2017; Chen et al., 2011; Endo et al., 2021; Graber et al., 2024; Kobayashi and Shinomoto, 2024; Masud and Borisyuk, 2011; Okatan et al., 2005). While these conditions are not always met in practice, they increase the reliability of synaptic connectivity inferred from functional interactions.

One limitation of the proposed approach is that while the spatial GLM successfully identified distance-based interactions generated from exponential, half-Gaussian, linear, and inverse square functions, it confounded linear and lognormal functions. Therefore, we did not include these functions in the analysis of calcium imaging data. As a result, we could not assess the fit of these functions and cannot rule out the possibility that other functions not considered here may offer a more accurate characterization of cortical interactions. Ultimately, however, experimentalists may be more interested in how these interactions are altered across layers (Senzai et al., 2019), cell types (Khoury et al., 2025), brain regions (Horvát et al., 2016), and stimuli (Ohiorhenuan et al., 2010; Smith and Kohn, 2008; Yatsenko et al., 2015), particularly given that several distance-dependent functions have a similar shape at short distances.

Another consideration is that while the proposed MLE approach provides a useful framework for investigating spatially dependent interactions, it describes a global decay function across an entire network and cannot resolve individual interactions between pairs of neurons. This limitation turns out to be an advantage in large-scale networks where we aim to characterize a spatial decay function without having to estimate all pairwise connections. In addition, MLE is not designed to distinguish between spatial interactions driven by local recurrent connections versus those driven by external inputs to a neuronal population.

A future direction is to apply MLE to distinguish between the spatial scales of different neuronal subtypes, including pyramidal neurons as well as subclasses of inhibitory interneurons (parvalbumin, somatostatin, and vasopressin) (Fino and Yuste, 2011; Jiang et al., 2015; Tremblay et al., 2016). Characterizing subtype-specific spatial patterns would deepen our understanding of how local and longer-range interactions contribute to network function.

More broadly, the proposed framework may offer insights into neurodevelopmental and neurodegenerative disorders marked by alterations in spatial organization. For instance, Alzheimer′s disease is associated with synaptic loss and reduced connectivity density (DeKosky and Scheff, 1990), while conditions such as epilepsy and autism spectrum disorder involve distinct disruptions in spatial circuit structure (Stoner et al., 2014). Understanding these differences at a subtype-resolved spatial scale could help clarify the circuit-level mechanisms underlying such pathologies.

To conclude, the proposed MLE approach offers an efficient and accessible tool for investigating spatially dependent interactions in neuronal circuits. This analysis provides a promising avenue to deepen our understanding of how structural constraints shape neural dynamics. Further, it opens the door to characterizing the relation between spatial organization and dynamics in large-scale data obtained from healthy and pathological brain circuits.

## Materials and methods

### Maximum likelihood estimation in a linear Poisson model

To estimate λ_decay_ based on the spiking activity of a linear Poisson model with baseline firing rate *r_0_* and coupling strength α, a maximum likelihood estimator (MLE) was employed as follows. Assuming Bernoulli spike data *S*_*i*_(*t*) ∈ {0,1}, the probability of spiking is given by


(3) $$P\left(S_{i}\left(t\right)=1\right)=\sigma \left(h_{i}\left(t\right)\right)$$

where


$$h_{i}\,\left(t\right)=r_{0}+\sum_{j\neq i}\alpha \cdot S_{j}\,\left(t-1\right)\cdot \exp\left(-\lambda_{\text{decay}}\cdot d_{i\,j}\right)$$

assuming a large population of neurons, and σ(*x*)=1/(1 + *exp*⁡(−*x*)). The likelihood is


$${ℒ}_{M\,L\,E}\,\left(\lambda_{\text{decay}}\right)=\prod_{t=2}^{T}\prod_{i=1}^{N}\sigma \,{\left(h_{i}\,\left(t\right)\right)}^{S_{i}\,\left(t\right)}\cdot {\left(1-\sigma \,\left(h_{i}\,\left(t\right)\right)\right)}^{1-S_{i}\,\left(t\right)}$$

Taking the log-likelihood,


(4) $$\log{ℒ}_{M\,L\,E}\left(\lambda_{\text{decay}}\right)=\sum_{t=2}^{T}\sum_{i=1}^{N}[S_{i}\left(t\right)\log\left(\sigma \left(h_{i}\left(t\right)\right)\right)+$$

$$\left(1-S_{i}\left(t\right)\right)log\left(1-\sigma \left(h_{i}\left(t\right)\right)\right)]$$

Defining *P*_*i*_(*t*)=σ(*h*_*i*_(*t*)) and applying the chain rule, the derivative of Equation 4 is obtained as


$$\frac{d}{d\,\lambda_{\text{decay}}}\,\log{ℒ}_{M\,L\,E}\,\left(\lambda_{\text{decay}}\right)=$$

$$\sum_{t=2}^{T}\sum_{i=1}^{N}\left[\frac{S_{i}\,\left(t\right)-P_{i}\,\left(t\right)}{P_{i}\,\left(t\right)\,\left(1-P_{i}\,\left(t\right)\right)}\cdot \frac{d\,P_{i}\,\left(t\right)}{d\,\lambda_{\text{decay}}}\right]$$

with the full derivative given by


$$\frac{d}{d\,\lambda_{\text{decay}}}\log{ℒ}_{M\,L\,E}\left(\lambda_{\text{decay}}\right)=-\alpha \sum_{t=2}^{T}\sum_{i=1}^{N}\left(S_{i}\left(t\right)-P_{i}\left(t\right)\right)\cdot$$

$$\sum_{j\neq i}S_{j}\,\left(t-1\right)\,d_{i\,j}\cdot e^{-\lambda_{\text{decay}}\,d_{i\,j}}$$

Practically, finding the best-fitting λ_decay_, denoted $\lambda_{\text{decay}}^{*}$, involves numerically minimizing the negative log-likelihood:


$$\lambda_{\text{decay}}^{*}=a\,r\,g\,\min_{\lambda_{\text{decay}}}-\log{ℒ}_{M\,L\,E}\,\left(\lambda_{\text{decay}}\right)\;$$

using gradient descent. We assume that the log-likelihood function (Equation 4) is maximized at the true parameter λ_decay_ employed to generate spike trains, which holds for long recordings (*T*→∞) given the law of large numbers. We further assume that the parameter λ_decay_ is identifiable, meaning that distinct values of λ_decay_ lead to distinct distributions of the observed data. This holds true given that λ_decay_ uniquely determines the distance-dependent interactions, and thus spike probabilities. Under reasonable conditions of smoothness for *ℒ*_*MLE*_(λ_decay_) and finite Fisher information, the MLE is asymptotically normal and converges in probability to the true parameter λ_decay_.

### Maximum likelihood estimation: exponential function

The MLE approach employed to estimate λ_decay_ from spike trains was based on the same method as described above for Poisson neurons but adapted to a scenario where only the spike raster is known, and not the baseline rate or the interaction strength between neurons. The log likelihood *ℒ*_*MLE*_ is the joint probability of observing the spiking activity given λ_decay_ (Equation 4). Given that the baseline firing rate and coupling strength for the Izhikevich model and calcium imaging data are assumed to be unknown, we substituted the spike probability of Equation 3 for


$$P\left(S_{i}\left(t\right)=1\right)\frac{I_{i}\,\left(t\right)}{N}$$

where *N* is the number of neurons. The interaction term *I*_*i*_(*t*) was computed as


$$I_{i}\,\left(t\right)=\sum_{j}S_{j}\,\left(t\right)\,W\,\left(i,j\right)$$

where


(5) $$W\,\left(i,j\right)=e^{-\lambda_{\text{decay}}\,d_{i\,j}}$$

and *S*_*j*_(*t*) is the spike activity of the presynaptic neuron *j* at time *t*. Finding the best-fitting λ_decay_ involves numerically minimizing the negative log-likelihood.

### Alternative functions for maximum likelihood estimation

In the case of a half-Gaussian function, *W*(*i*,*j*) (Equation 5) is replaced with


$$W\,\left(i,j\right)=e\,x\,p\,\left(-\frac{d_{i\,j}^{2}}{2\,\sigma^{2}}\right)$$

where we aim to estimate σ (the width of the half-Gaussian function). The log likelihood function is the same as before. We can also substitute in a log-normal function:


$$W\,\left(i,j\right)=\frac{1}{d_{i\,j}\,\sigma \,\sqrt{2\,\pi }}\,e\,x\,p\,\left(-\frac{{\left(\log d_{i\,j}^{2}-\mu \right)}^{2}}{2\,\sigma^{2}}\right)$$

with dimensionless parameters σ and μ. To focus on estimating σ, we fix μ as follows:


$$\mu =\frac{1}{N\,\left(N-1\right)}\,\sum_{i\neq j}l\,o\,g\,\left(d_{i\neq j}\right)$$

For a linear distance-dependent function,


$$W\,\left(i,j\right)=1-\sigma \,d_{i\,j}$$

and for an inverse square function,


$$W\,\left(i,j\right)=\frac{1}{d_{i\,j}^{2}+\sigma }$$

### Spatial generalized linear model

For the spatial GLM, the expression for the spike probability *P*(*S*_*i*_(*t*)=1) follows Equation 3. The *log*⁡*ℒ*_*GLM*_(λ_decay_,α,*r*_0_) follows Equation 4, but is estimated over three parameters instead of only λ_decay_. Gradients for these parameters are obtained as follows,


$$\frac{\partial {ℒ}_{G\,L\,M}}{\partial r_{0}}=\sum_{t=2}^{T}\sum_{i=1}^{N}\left(S_{i}\,\left(t\right)-P_{i}\,\left(t\right)\right)$$

$$\frac{\partial {ℒ}_{G\,L\,M}}{\partial \alpha }=\sum_{t=2}^{T}\sum_{i=1}^{N}\left(S_{i}\,\left(t\right)-P_{i}\,\left(t\right)\right)\cdot h_{i}\,\left(t\right)$$

$$\frac{\partial {ℒ}_{G\,L\,M}}{\partial \lambda_{\text{decay}}}=\sum_{t=2}^{T}\sum_{i=1}^{N}\left(S_{i}\,\left(t\right)-P_{i}\,\left(t\right)\right)\cdot g_{i}\,\left(t\right)$$

where


$$g_{i}\,\left(t\right)=\sum_{j\neq i}S_{j}\,\left(t-1\right)\cdot \left[-\alpha \,d_{i\,j}\,\exp\left(-\lambda_{\text{decay}}\,d_{i\,j}\right)\right]$$

The spatial GLM captures the spatiotemporal dynamics of the system, including time-dependence and directional interactions (i.e., from neuron *j* to *i*). By comparison, MLE is not a full generative model — instead, summary statistics are extracted from the spike raster as a whole, then a spatially-dependent function, such as an exponential decay, is fitted to capture how functional connectivity declines with spatial distance.

### Transfer entropy

Transfer entropy is a powerful information-theoretic measure used to quantify directed interactions between time series (Izzi et al., 2024; Kajiwara et al., 2021; Lizier et al., 2008; Lungarella et al., 2007; Novelli et al., 2019; Schreiber, 2000; Ursino et al., 2020; Verdes, 2005; Vicente et al., 2011; Voges et al., 2024). It quantifies causal influences by assessing the degree to which the past activity of one neuron (potentially pre-synaptic) improves the predictability of the future activity of another neuron (potentially post-synaptic) (Kobayashi and Shinomoto, 2024; Vicente et al., 2011). Given two neurons *X* (source) and *Y* (target), transfer entropy is calculated as:


$$T\,E_{X\to Y}\,\left(△\,t\right)=$$

$$\sum_{y_{t+1,y_{t}^{\left(k\right)},x_{t}^{\left(l\right)}}}p\,\left(y_{t+1},y_{t}^{\left(k\right)},x_{t}^{\left(l-△\,t\right)}\right)\,l\,o\,g\,\frac{p\,\left(y_{t+1}|y_{t}^{\left(k\right)},x_{t}^{\left(l-△\,t\right)}\right)}{p\,\left(y_{t+1}|y_{t}^{\left(k\right)}\right)}$$

where *y*_*t+1*_ is the state of the target neuron at the next time step, $y_{t}^{k}$ are the past states of the target neuron *Y* up to *k* time steps, $x_{t}^{\left(l-△\,t\right)}$ are the past states of the source neuron up to *l*−△*t* time steps, and △*t* is a time delay (set to 1 ms).

### Granger causality

To compute Granger causality for a pair of neurons, we assumed two spike trains *X*(*t*) and *Y*(*t*) over a time window [0,*T*] and binned them using bins of size △*t* = 10 ms. We then chose a history window *ℋ* = 100 ms and for each time *t*, create covariates using the past spiking activity of both neurons:


$${\text{h}}_{X}\left(t\right)=\left[x_{t-1},x_{t-2},,x_{t-ℋ}\right]$$

$${\text{h}}_{Y}\left(t\right)=\left[y_{t-1},y_{t-2},,y_{t-ℋ}\right]$$

Then, two models were fitted to represent the instantaneous probability of neuron *X*. A reduced model based only on the history of *X* is provided by


$$\log\lambda_{X}^{r\,e\,d\,u\,c\,e\,d}\,\left(t\right)=\mu_{X}+{\text{w}}_{X\,X}\,{\text{h}}_{X}\,\left(t\right)$$

and a full model combining the history of *X* and *Y* is given by


$$\log\lambda_{X}^{f\,u\,l\,l}\,\left(t\right)=\mu_{X}+{\text{w}}_{X\,X}\,{\text{h}}_{X}\,\left(t\right)+{\text{w}}_{Y\,X}^{T}\,{\text{h}}_{Y}\,\left(t\right)$$

where μ_*X*_ is the mean firing rate of neuron *X*, and **w**_*XX*_ and ${\text{w}}_{Y\,X}^{T}$ are weights to be estimated. Both models were fitted numerically using maximum likelihood estimation. The likelihood of the reduced model is given by


$${ℒ}_{r\,e\,d\,u\,c\,e\,d}=\sum_{t}\left[x\,\left(t\right)\cdot \log\left(\lambda_{X}^{r\,e\,d\,u\,c\,e\,d}\,\left(t\right)\cdot \Delta \right)-\lambda_{X}^{r\,e\,d\,u\,c\,e\,d}\,\left(t\right)\cdot \Delta \right]$$

where Δ is the bin width. Similarly, for the full model,


$${ℒ}_{f\,u\,l\,l}=\sum_{t}\left[x\,\left(t\right)\cdot \log\left(\lambda_{X}^{f\,u\,l\,l}\,\left(t\right)\cdot \Delta \right)-\lambda_{X}^{f\,u\,l\,l}\,\left(t\right)\cdot \Delta \right]$$

The strength of causal interaction is given by


$$G\,C_{Y\to X}=\frac{{ℒ}_{f\,u\,l\,l}-{ℒ}_{r\,e\,d\,u\,c\,e\,d}}{\left|{ℒ}_{r\,e\,d\,u\,c\,e\,d}\right|}$$

which yields a unitless ratio of how much better the model performs at predicting the activity of neuron *X* by including the history of neuron *Y*.

### Izhikevich model

A network of Izhikevich neurons with *N* = 1,000 excitatory neurons was simulated (Figure 3A). The dynamics of each neuron’s membrane potential (*v_i_*) and recovery variable (*u_i_*) are described by


$$\frac{d\,v_{i}}{d\,t}=0.04\,v_{i}^{2}+5\,v_{i}+140-u_{i}+I_{i}$$

$$\frac{d\,u_{i}}{d\,t}=a_{i}\,\left(b_{i}\,v_{i}-u_{i}\right)$$

Spikes are emitted when *v*_*i*_=*v*_*thresh*_, where *v*_*thresh*_ = 30 mV. Then, the membrane potential and recovery variable are reset to *v*_*i*_←*c*_*i*_ and *u*_*i*_←*u*_*i*_ + *d*_*i*_, where *a_i_*, *b_i_*, *c_i_*, and *d_i_* are parameters set as follows: *a_i_* = 0.02, *b_i_* = 0.2, *c_i_* = −65, and *d_i_* = 8. The input current is


$$I_{i}^{s\,y\,n}=\sum_{j=1}^{N}J_{i\,j}\,S_{j}$$

where *J*_*ij*_ is the synaptic weight from neuron *j* to *i*, and *S_j_* = 1 if *v*_*i*_=*v*_*threshold*_, and *S_j_* = 0 otherwise. The external input is $I_{i}^{e\,x\,t}=𝒩\,\left(\mu ,\sigma^{2}\right)$ and the total input is $I_{i}=I_{i}^{s\,y\,n}+I_{i}^{e\,x\,t}$. Each neuron *i* was assigned a position **x**_*i*_=(*x*_*i*_,*y*_*i*_) in two-dimensional space, and the probability of pairwise connections decayed exponentially with distance (Equation 1), with *c* = 30. Sparse connectivity was generated by connecting any given pair of neurons with a 10% probability, while the remainder of weights were set to zero. While inhibitory neurons are beyond our scope, they could be incorporated in further refinements of the model.

Using mean field theory, we define the mean firing rate of the network as


$$r=\left\langle S_{j}\,\left(t\right)\right\rangle$$

where ⟨⋅⟩ denotes averaging over time and neurons. We then replace the exact spike trains with their statistical averages,


$$I_{i}^{s\,y\,n}\,\left(t\right)\approx J\,\sum_{j}S_{j}\,\left(t\right)\approx J\,N\,r$$

where *J* is the mean synaptic strength and *N* is the number of presynaptic neurons. Synaptic fluctuations are approximated by a stationary Gaussian process with mean *JNr* and variance *J*^2^*r*. Thus, the effective input current follows


$$I_{i}\,\left(t\right)\approx J\,N\,r+\eta_{i}\,\left(t\right)$$

where η_*i*_(*t*) is a Gaussian noise term with variance


$$V\,a\,r\,\left(\eta_{i}\right)=J^{2}\,r+\sigma^{2}$$

Let us consider two neurons *i* and *j* in the network. These neurons receive inputs from a subset of common presynaptic neurons. Let *S*_*ij*_ denote the fraction of presynaptic neurons shared by both *i* and *j*. The total input currents are:


$$I_{i}=\sum_{k\in p\,r\,e\,\left(i\right)}J\,S_{k}$$

where *pre*(*i*) denotes the set of presynaptic neurons projecting to *i* and *j*. The fraction of shared presynaptic inputs between neurons *i* and *j* is given by


$$K_{i\,j}=\frac{\left|p\,r\,e\,\left(i\right)\,p\,r\,e\,\left(j\right)\right|}{\left|p\,r\,e\,\left(i\right)\right|}$$

The shared presynaptic inputs introduce correlations in the inputs *I_i_* and *I_j_*. The covariance of inputs between neurons *i* and *j* is


$$C\,o\,v\,\left(I_{i},I_{j}\right)\approx J^{2}\,r\,K_{i\,j}$$

with variance


$$V\,a\,r\,\left(I_{i}\right)=V\,a\,r\,\left(I_{i}\right)=J^{2}\,r+\sigma^{2}$$

The correlation coefficient is given by


$$C_{i\,j}=\frac{C\,o\,v\,\left(I_{i},I_{j}\right)}{\sqrt{V\,a\,r\,\left(I_{i}\right)\,V\,a\,r\,\left(I_{j}\right)}}$$

Thus, the correlation simplifies to


$$C_{i\,j}=\frac{J^{2}\,r\,K_{i\,j}}{J^{2}\,r+\sigma^{2}}$$

For weak coupling, we can approximate the correlation as


(6) $$C_{i\,j}\approx \frac{J^{2}}{J^{2}+\sigma^{2}}\,K_{i\,j}$$

Assuming a homogeneous spatial network where the shared input fraction decays exponentially with distance,


$$K_{i\,j}\approx e^{-2\,\lambda_{\text{decay}}\,d_{i\,j}}$$

we can rewrite Equation 6 as


$$C_{i\,j}\approx \frac{J^{2}}{J^{2}+\sigma^{2}}\,e^{-2\,\lambda_{\text{decay}}\,d_{i\,j}}$$

Given that neurons receive synaptic inputs from a common pool of presynaptic neurons, the fraction of shared inputs between two neurons separated by distance *d* is approximately


$$K_{i\,j}\approx \int_{0}P\,\left(d\right)\,e^{-\lambda_{\text{decay}}\,d}\,dd=e^{-2\,\lambda_{\text{decay}}\,d}$$

We can then rewrite the approximate correlations as


$$C_{i\,j}\approx \frac{J^{2}\,e^{-2\,\lambda_{\text{decay}}\,d}}{J^{2}\,e^{-2\,\lambda_{\text{decay}}\,d}+\sigma^{2}}$$

As λ_decay_→0, connectivity becomes all-to-all, hence *C*_*ij*_ approaches its upper bound,


$$C_{i\,j,\lambda_{\text{decay}}\to 0}\to \frac{J^{2}}{J^{2}+\sigma^{2}}$$

Conversely, when λ_decay_ is large, shared inputs become negligible, hence *C*_*ij*_→0.

### Calcium data

Activity from mouse V1 cortex was obtained using two-photon calcium imaging. Details are found in related work (Stringer et al., 2019a). Each recording site measured 12 × 12 μm^2^, and the vertical spacing between adjacent sites was 20 μm. The recording area was 0.9 mm by 0.935 mm, with plane depth of 0.35 mm. Spike were extracted using OASIS (Friedrich et al., 2017; Pachitariu et al., 2018).

## Data Availability

Publicly available datasets were analyzed in this study. These data can be found here: https://janelia.figshare.com/articles/dataset/Recordings_of_ten_thousand_neurons_in_visual_cortex_during_spontaneous_behaviors/6163622.
