# Supplementary material for: Maximum likelihood estimation of spatially dependent interactions in large populations of cortical neurons
Source: Front Comput Neurosci. 2025 Aug 13;19:1639829. doi: 10.3389/fncom.2025.1639829 (PMC12380633; doi:10.3389/fncom.2025.1639829)
Supplement: Supplementary file 1 [file Data_Sheet_1.pdf]

Supplementary Material

MLE approximation of distance-dependent functions

Simulations of the linear Poisson model were performed across different distance-dependent interactions characterized by an exponential, half-Gaussian, linear, lognormal, and inverse square function (see Materials and Methods). While the approximation of an exponential function is provided in the main paper (**Fig.2C-D**), here we provide results related to the alternative functions (**Fig.S1**). The MLE provided an accurate fit to these functions, with the exception of the inverse square where the approximation was poorer. Overall, the ability of MLE to estimate distance-dependent interactions based on spike data extended to several functions characterizing the statistical dependency between spikes and inter-neuronal distance.

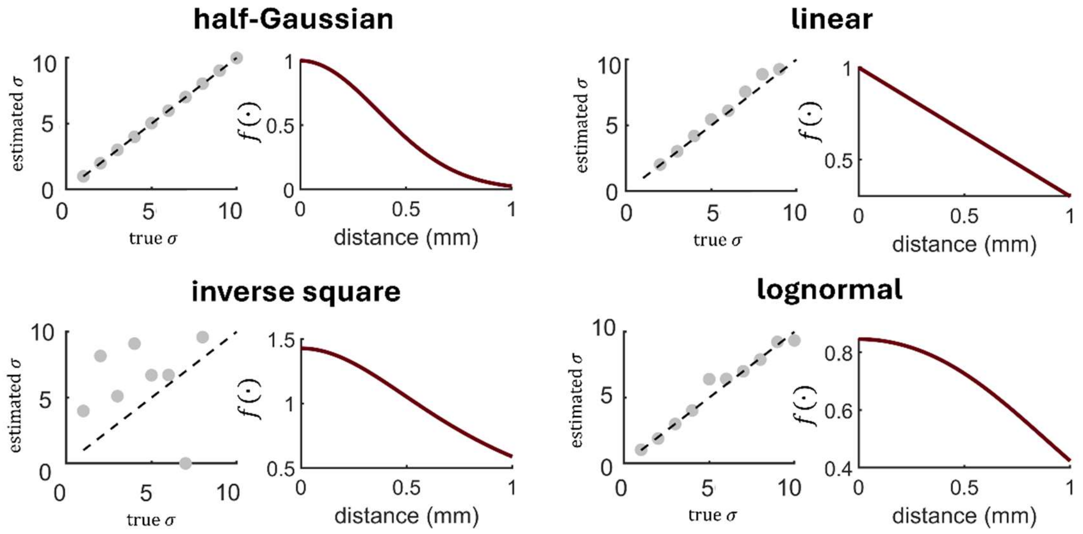

Figure S1. Maximum likelihood estimates of σ across different runs of the Poisson model with various distance-dependent functions. Dashed line is a unity line showing an exact correspondence between the true and estimated values of σ. Solid red lines show examples of functions including half-Gaussian (σ=0.7 mm), linear (σ=0.7 mm^{-1}), inverse square (σ=0.7 mm^2), and lognormal (σ=0.7, μ=-1.5).

Sources of MLE bias

Using simulations of linear Poisson neurons, we examined several factors that influence the estimation of distance-based interactions. First, MLE error decreased as the number of neurons (**Fig.S2A**), duration of simulation (**Fig.S2B**), and firing rates (**Fig.S2C**) increased. Stable estimates required with a minimum of approximately 100 neurons, 1,000 ms of activity, and a mean rate of 2 Hz.

Next, we examined the effect of subsampling a subset of neurons amongst a larger population. Subsampling is typical of experimental recordings, where activity from only a small proportion of all

neurons is recorded. In simulations with 1,000 neurons, MLE error decreased as the subset of neurons increased (**Fig.S2D-E**). MLE accuracy thus favored denser sampling when estimating spatially dependent interactions. The relation between sampling and error was approximately linear across a broad range of subsampling values.

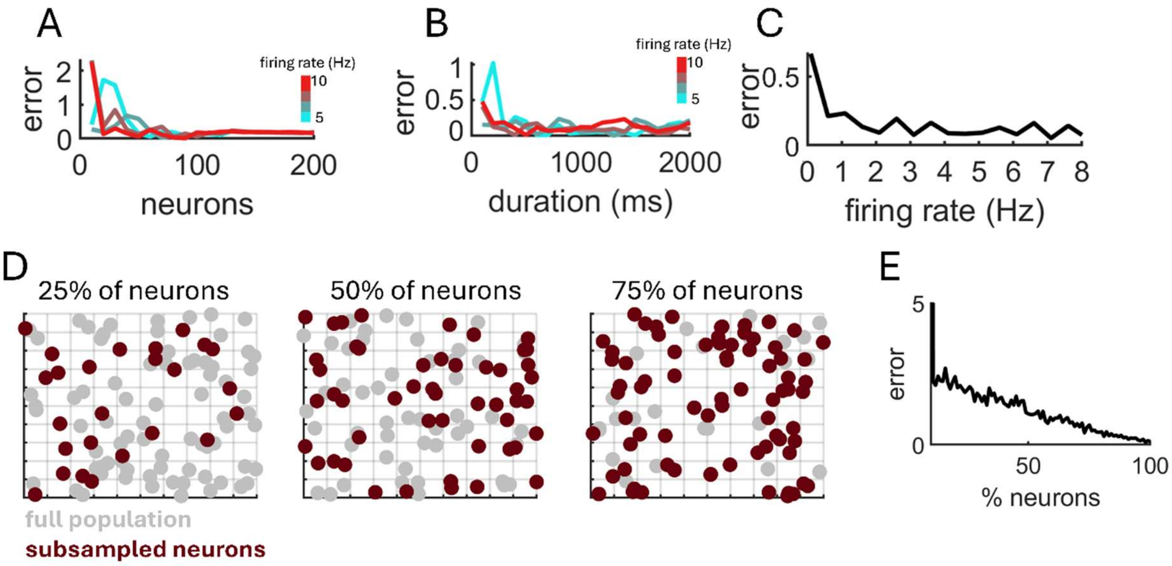

Figure S2. MLE estimation error. (**A**) Error decreases as the number of simulated neurons increases. (**B**) Duration of spike data. (**C**) Firing rate. (**D**) Spatial layout of a population of neurons when subsampling 25%, 50%, and 75% of neurons. (**E**) Increasing the percentage of subsampled neurons decreases MLE error in an approximately linear fashion.

Because modeled neurons were embedded on a two-dimensional sheet, we aimed to rule out boundary effects caused by finite edges. This was achieved by randomly distributing neurons on a toroidal space (**Fig.S3A**), which has no finite edges. MLE estimation of spatial interactions were accurate across a range of λ_{decay} values (**Fig.S3B**). Another way to address boundary effects is to consider the impact of restricting the placement of neurons to a finite domain size within a two-dimensional sheet (**Fig. S3C**). Smaller domain sizes resulted in larger MLE error, given that spatial interactions are more difficult to resolve when neurons are tightly packed (**Fig. S3D**). Domain sizes of 250 µm and above yielded consistently low MLE error, indicating that a minimum domain size is required for an accurate estimation of distance interactions.

Another factor contributing to MLE accuracy is the introduction of synaptic transmission delays between pairs of modeled neurons. A uniform distribution of delays in the range of 1-5 ms, as typical within a delimited cortical area (1 mm^3), was introduced between all pairs of simulated neurons (**Fig.S4A**) and MLE was applied on the resulting spike trains to yield estimates of λ_{decay} (**Fig.S4B**). Estimation error rose in an approximately linear fashion as the range of delays was increased (from 1-5 ms to 1-6 ms, 1-7 ms, etc.) (**Fig.S4C**). Increasing the range of delays did not have a visible impact on the overall dynamics of neural activity across the population (**Fig.S4D**).

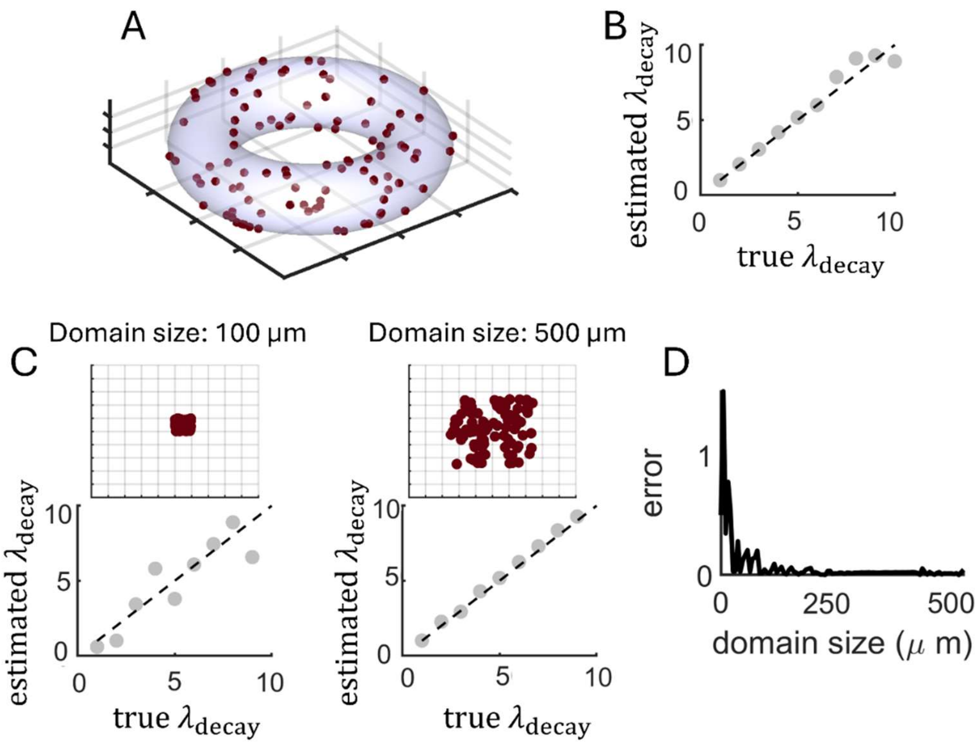

Figure S3. Boundary and domain-size effects in estimating distance-dependent interactions. (**A**) Distribution of neurons on a torus topology. (**B**) Estimation of distance-dependent interactions on a torus. (**C**) Impact of domain size. Top: placement of neurons on a restricted domain. Bottom: MLE estimation. (**D**) Estimation error decreases as domain size increases.

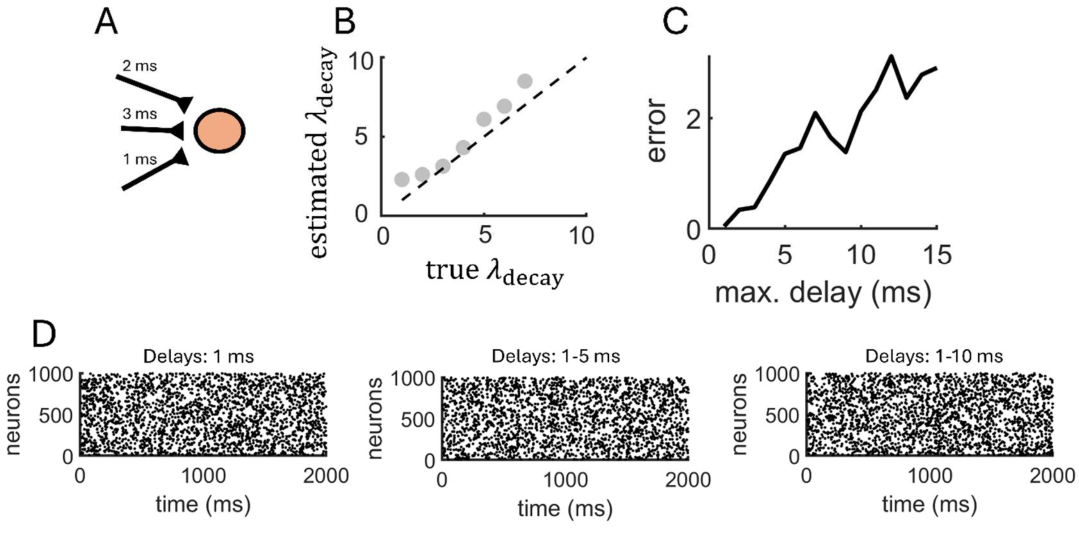

Figure S4. Impact of synaptic delays on MLE estimation. (**A**) Introducing delays in the Poisson model. (**B**) MLE estimation with delays sampled from a uniform distribution in the range of 1-3 ms. (**C**) Increasing the maximum value in the distribution of delays leads to higher estimation error. (**D**) Examples of rasters obtained with different ranges of synaptic delays.

__Distance-dependent interactions in calcium imaging data__

We characterized distance-dependent interactions in calcium imaging data using spike-count correlations, transfer entropy, and Granger causality. Results are reported as grand averages across all datasets, as well as averages across datasets of spontaneous and evoked activity separately (**Fig.S5**). Spike count correlations and transfer entropy show a consistent decrease in the strength of interactions as pairwise distances increase, with a less pronounced effect obtained for Granger causality.

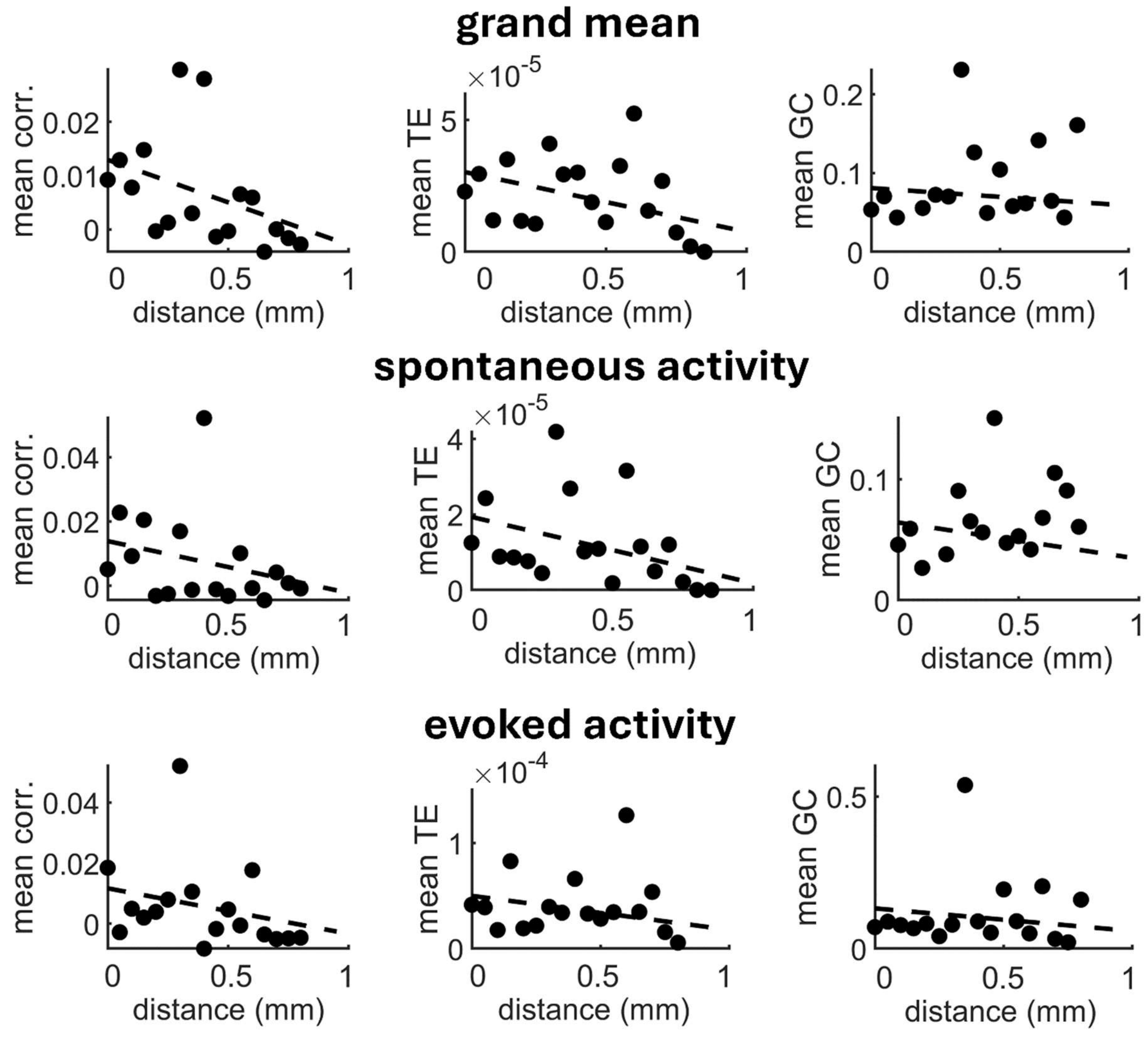

s

Figure S5. Distance-based interactions in two-photon calcium imaging data. “mean corr.” are average spike-count correlations (bins of 10 ms). “TE”: transfer entropy. “GC”: Granger causality. The grand mean is obtained across all 14 datasets. Spontaneous activity is obtained from 9 datasets and evoked activity is obtained from 5 datasets. Dashed line: best-fitting regression.
